# Prevalence and differences of ideal cardiovascular health in urban and rural adolescents in the Region of Tyrol: results from the EVA Tyrol study

**DOI:** 10.1186/s12872-021-02156-6

**Published:** 2021-07-13

**Authors:** C. Hochmayr, J. P. Ndayisaba, N. Gande, A. Staudt, B. Bernar, K. Stock, R. Geiger, M. Knoflach, U. Kiechl-Kohlendorfer, Early Vascular Ageing (EVA) Study Group, Mandy Asare, Manuela Bock-Bartl, Maximilian Bohl, Christina Schreiner, Gregor Brössner, Tatjana Heisinger, Julia Klingenschmid, Martina Kothmayer, Julia Marxer, Raimund Pechlaner, Maximilian Pircher, Carmen Reiter, Sophia Zollner-Kiechl, Stefan Kiechl, Bernhard Winder

**Affiliations:** 1 Department of Pediatrics II (Neonatology), Medical University of Innsbruck, Anichstraße 35, 6020 Innsbruck, Austria; 2 Department of Neurology, Medical University of Innsbruck, Anichstraße 35, 6020 Innsbruck, Austria; 3 Department of Pediatrics I, Medical University of Innsbruck, Innsbruck, Austria; 4 Department of Pediatrics III (Cardiology), Medical University of Innsbruck, Innsbruck, Austria; 5 Medical University of Innsbruck, Innsbruck, Austria

**Keywords:** Cardiovascular health, Epidemiology, Public health, Urban rural, Adolescents, Risk factors

## Abstract

**Purpose:**

Early adoption of a healthy lifestyle has positive effects on cardiovascular health (CVH) in adulthood. In this study, we aimed to assess CVH metrics in a cohort of healthy teenagers with focus on differences between rural and urban areas.

**Methods:**

The Early Vascular Aging (EVA) Tyrol study is a population-based non-randomized controlled trial, which prospectively enrolled 14- to 19-year-old adolescents in North Tyrol, Austria and South Tyrol, Italy between 2015 and 2018. Data from the baseline and control group (prior to health intervention) are included in the current analysis. CVH determinants (smoking, body mass index, physical activity, dietary patterns, systolic and diastolic blood pressure, total cholesterol and fasting blood glucose) were assessed and analyzed for urban and rural subgroups separately by univariate testing. Significant variables were added in a generalized linear model adjusted for living in urban or rural area with age and sex as covariates. Ideal CVH is defined according to the guidelines of the American Heart Association.

**Results:**

2031 healthy adolescents were enrolled in the present study (56.2% female, mean age 16.5 years). 792 adolescents (39.0%) were from urban and 1239 (61.0%) from rural areas. In 1.3% of adolescents living in urban vs. 1.7% living in rural areas all CVH determinants were in an ideal range. Compared to the rural group, urban adolescents reported significantly longer periods of moderate to vigorous-intensive activity (median 50.0 min/day (interquartile range 30–80) vs. median 40.0 min/day (interquartile range 25–60), p < 0.01). This observation remained significant in a generalized linear model (p < 0.01). There were no significant differences between the study groups regarding all other CVH metrics.

**Conclusion:**

The low prevalence of ideal CVH for adolescents living in urban as well as rural areas highlights the need for early health intervention. Geographic differences must be taken into account when defining targeted subgroups for health intervention programs.

**Supplementary Information:**

The online version contains supplementary material available at 10.1186/s12872-021-02156-6.

## Introduction

Cardiovascular disease (CVD) is on top of the leading causes of death globally [1]. According to the WHO an estimated 17.9 million people died from CVD in 2016 worldwide, of which more than three quarters were due to myocardial infarction and stroke [2]. In Europe, CVD claimed 3.9 million lives, which corresponds to 45% of all deaths [3]. There is evidence that early atherosclerotic vessel alterations can already occur early in life. Autopsy studies in young individuals clearly documented a strong relation between cardiovascular risk factors and vessel wall lesions. Initiation of inflammatory processes like local accumulation of T-cells may trigger the development of fatty streaks which have been identified as early stages of atherosclerosis [4–6].

Early adoption of a healthy lifestyle has positive effects on CVH in adulthood [1, 7]. In order to measure and promote CVH in adolescents and young adults the American Heart Association (AHA) developed the concept of seven health metrics including four health behaviors (non-smoking, ideal Body Mass Index (BMI), regular physical activity (PA), favorable dietary patterns) and three health factors (blood pressure (BP), total cholesterol and fasting blood glucose). These parameters correspond to positive formulation of the modifiable risk factors of CVD [7]. Recently, several studies proved that the higher ideal CVH level was in adolescence and young adulthood the lower was the risk of CVD later in life [8, 9]. Furthermore, improvement of childhood risk factors like obesity or hypertension leads to a lower incidence of CVD and has a positive impact on public health. Still, the exact mechanisms of how childhood health behaviors like healthy diet and PA contribute to adulthood CVD remain unclear and need to be further investigated [10].

Previous studies in adults indicated that health behavior and consequently CVH differ between urban and rural areas. [11, 12]. Recently, a systematic review based on 2009 population-based studies demonstrated that over the last 33 years rising BMI in adults living in rural areas mainly contributed to the worldwide increase of obesity and overweight [13]. So far, most of the studies investigating the relation between CVD risk factors and place of living have focused on the adult population [14, 15]. However, environmental factors also influence the lifestyle of adolescents and must be considered in health promotion and the design of intervention programs for the youth.

Therefore, we investigated the prevalence of ideal CVH and compared CVH metrics according to living in an urban or rural area in a cohort of healthy adolescents in the federal province of North Tyrol, Austria, and South Tyrol, Italy. To the best of our knowledge, we are the first to investigate urban–rural differences in CVH in a large teenage population in central Europe.

## Methods

### Study design and population

This study is part of a clinical trial, the Tyrolean Early Vascular Aging-Study (EVA Tyrol) that was conducted in Tyrol, a geographically defined region in the Western part of Austria consisting of 745,000 inhabitants and in Bruneck, a city in the Autonomous Province of Bolzano-South Tyrol, Italy with around 80,000 inhabitants between May 2015 and July 2018.

In order to achieve a homogenous local and social distribution invitations to participate in the project were sent to all schools in Tyrol as well as large Tyrolean companies with a target population of 14- to 19-year-old adolescents. Apart from missing written informed consent or absence on the day of examination there were no exclusion criteria. All included participants gave written informed consent and in case participants were younger than the age of 18, a written informed consent was also signed by the parents or the legal guardian. Study participants were assigned to either a health intervention or a control group. Those assigned to the health intervention group received a baseline examination and were invited to a follow-up examination after two years. Within the two-year interval they were offered a special health intervention program. In order to proof the effectiveness of the health intervention program by comparing the prevalence of the AHA health metrics in both arms a control group underwent the baseline examination without getting the health intervention. The current evaluation included all participants without health promotion (i.e. baseline examination and control group).

Detailed information regarding recruitment of participants, data collection, and health intervention program has been previously published [16].

The study was performed in accordance with the Declaration of Helsinki, ethical approval was granted from the review board of the Medical University of Innsbruck, Austria (approval number AN 2015-0005 345/4.13). The study is registered at www.clinical.trials.gov (NCT number 03929692), first registration on 29/04/2019.

### Assessment of lifestyle risk factors

Behavioral health components were assessed within the scope of a standardized case report form adopted from the Bruneck Study [17], Atherosclerotic Risk Factors in Male Youngsters- [6], Atherosclerotic Risk Factors in Female Youngsters- [18] and Health Behaviour in School-aged Children Survey [19] studies and a personal interview conducted by the study staff in attendance of a specialist in Pediatrics.

According to the definitions of the AHA and our standardized questionnaire individuals were defined as smokers if they had smoked in the last 30 days at the time of the interview or reported regular tobacco use of at least one cigarette per week, all the others were categorized ideal regarding smoking status [17].

Healthy diet was recorded by a score based on the Dietary Approaches to Stop Hypertension- (DASH-) diet [20] consisting of five favorable components of which one point could be scored for each; frequency of fruit and vegetables (at least four to five servings per day); frequency of fish meals (at least two servings per week, each 100 g); frequency of whole-grain products (at least three servings per day, each about 30 g); amount of salt (less than 1.5 g per day); amount of sugar-sweetened drinks (maximum of one liter per week, max. 450 kcal per week).

PA was self-reported in a standardized interview and documented as the average count of minutes of moderate- or vigorous-intensity activity per day. Brisk walking served as the comparative basis for moderate-intensity activity and running or breathing rapidly during PA for vigorous-intensity activity.

### Anthropometry

Anthropometric measurements included weight, determined by means of calibrated medical precision scale, and height measured without shoes using a Harpenden stadiometer. BMI was calculated as weight in kilograms divided by height in meters squared (kg/m^2^) and converted to percentiles according to data by Kromeyer-Hauschild et al. [21].

BP measurement was performed with an automated oscillometric device Intelli Sense (OMRON M4-I Healthcare Co., Kyoto, Japan). Values were taken on the left or right upper arm on seated subjects after at least 5 min at rest using an appropriate cuff size for three times from which the mean value was calculated. Based on a reference data set the mean BP values were assigned to corresponding percentiles [22].

### Laboratory blood testing

Blood samples were drawn at the beginning of the examination after a fasting period of more than 8 h from a cubital vein. Samples were immediately stored in cooling boxes (approximately 4 °C) and transported to the testing facility (iso-certified central institute for medical and chemical laboratory- diagnostics – ZIMCL, Tirol Kliniken, Innsbruck, Austria). Blood serum was used to determine levels of fasting blood glucose with the hexokinase enzymatic method, and total cholesterol with enzymatic colorimetric assay. Both parameters were analyzed with Cobas 8000 apparatus using Roche reagents.

### Definition of ideal CVH

Except for smoking every health metric was categorized in ideal, intermediate and poor. Table 1 summarizes the definition of health lifestyle according to the AHA [7].

**Table 1:** Categories ideal, intermediate and poor of the seven AHA health metrics according to Lloyed-Jones et al. [7]

Health metric	ideal	Intermediate	poor
BMI*	< 85th percentile	85th–95th percentile	> 95th percentile
BP^#^	< 90th percentile	90th–95th percentile (≥ 120 mmHg sys or ≥ 80 mmHg dia)	> 95th percentile
Fasting blood glucose	< 100 mg/dl	100–125 mg/dl	≥ 126 mg/dl
Total cholesterol	< 170 mg/dl	170–199 mg/dl	≥ 200 mg/dl
Smoking	has never smoked, has never smoked a whole cigarette		has smoked prior 30 days, regular tobacco usage
Healthy (DASH-) diet	4–5 components	2–3 components	0–1 components
PA	≥ 60 min/day	0–59 min/day	None

*BMI* Body Mass Index, *BP* blood pressure, *sys* systolic, *dia* diastolic, *DASH* Dietary Approaches to Stop Hypertension, *PA* physical activity

*BMI was converted to age and sex specific percentiles according to data by Kromeyer-Hauschild et al. [21]

^#^BP values were converted to age and sex specific percentiles according to the KiGGS Study [22]

### Assessing socio-economic status (SES)

SES was assessed by the Family Affluence Score (FAS). Three categories (low, middle and high affluence) are based on self-reported information about four status items. These include owning a car in the family, having an own bedroom, vacations and number of computers in the household [23]. In our cohort, low FAS occurred in no more than 1% so we combined low and middle affluence in one category. The two remaining categories (high and middle/low) were used to determine differences between rural and urban FAS.

### Defining urban and rural subgroups

Information on the place of living was derived from the case report form, where study participants had to state the zip code of their current main domicile. Classification into origin from the urban or rural site in the Austrian regions was conducted in accordance with the definitions of *Statistik Austria* [24]. The urban–rural typology of *Statistik Austria* combines international used typologies with the traditional Austrian classification into rural and urban areas according to population-density based factors as well as economic aspects (jobs, infrastructure facilities, commuter interrelations, accessibility to urban centers). South Tyrolean participants from the seven biggest cities with more than 10,000 inhabitants (Bozen, Meran, Brixen, Leifers, Bruneck, Eppan and Lana) were categorized urban, the rest was classified as rural.

### Statistical analysis

Data analyses were performed using the SPSS software version 26.0 for Windows (SPSS Inc., Chicago Illinois, USA). Data are presented as median (interquartile ranges) or mean ± SD and categorical variables as numbers (percentages). Differences in CVH metrics between rural and urban groups were determined using t-test or Mann–Whitney-U-test (depending on data distribution) and Pearson χ^2^- test (for categorical variables). The impact of living in rural or urban areas on CVH metrics (PA, BMI, systolic BP, diastolic BP, total cholesterol and fasting blood glucose) was assessed using a generalized linear model. Parameters entered in the respective generalized linear model were living in an urban or rural area, age and sex. Logistic regression was used, to assess the association between ideal CVH metrics and living in rural or urban. For this purpose a summary CVH score was calculated by adding all seven ideal CVH metrics from Table 1 (poor = 0, intermediate = 1 and ideal = 2) and entered in the model as quartiles (Quartile 1, CVH score ≤ 9; Quartile 2, CVH score = 10–11; Quartile 3, CVH score = 12; Quartile 4, CVH score = 13–14) with the lowest quartile (Quartile 1) as reference.

Parameters entered in all models were living in an urban or rural area, age and sex. P-values of less than 0.05 were considered statistically significant.

## Results

2102 students and trainees were enrolled in the EVA-study cohort representing about 5% of the eligible population in this age group in the region. 14 adolescents had to be excluded due to an age of 20 years or older and another 57 due to missing information on the place of living remaining 2031 cases for further analysis.

**Fig. 1:**
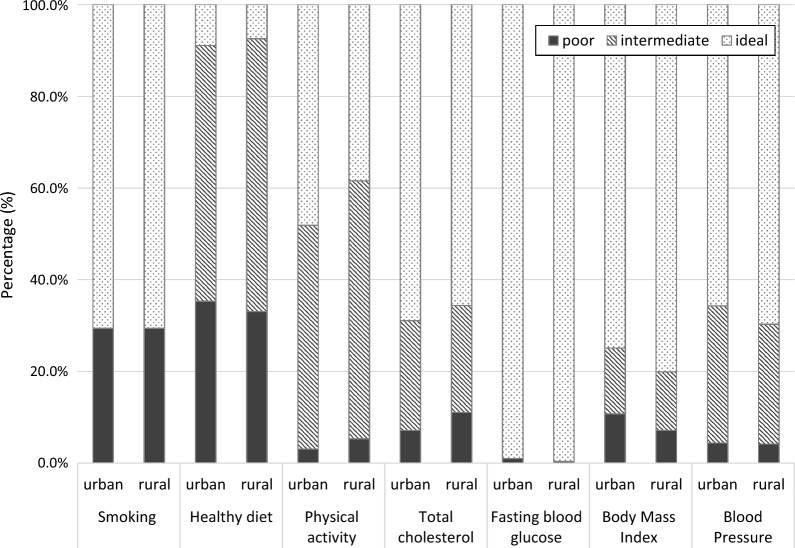
Poor, intermediate and ideal levels of each health metric according to urban and rural living. The x-axis shows each CVH metric by urban and rural living. In the y-axis the percentages of poor, intermediate and ideal CVH health for each health metric are given

Characteristics of the study population are displayed in Table 2. Mean age at examination was 16.5 years (SD 1.2), 1141 (56.2%) adolescents were girls. 1862 (88.6%) were students and 240 (11.4%) were trainees. 1239 (39.0%) participants resided in urban and 792 (61.0%) in rural areas. The prevalence of teenagers meeting criteria of all 7 ideal CVH determinants was generally low in our population. Only in 1.3% of adolescents living in urban vs. 1.7% living in rural areas all CVH determinants were in an ideal range (see Additional file 1: Figure S1 for detailed information). Figure 1 shows the proportions of poor, intermediate and ideal levels for each CVH metric according to urban and rural origin. Prevalence of ideal dietary habits was lower than 10% in both areas and about 30% of teenagers are classified as smokers. Furthermore, a high proportion of adolescents were categorized as intermediate concerning PA. No differences between the quartiles of the summary score of ideal CVH metrics in urban/rural living was found (p = 0.462 in univariate and p = 0.638 in multivariate analysis).

**Table 2:** Characteristics of the study population and association of health metrics with rural and urban lifestyle in univariate data analysis

Parameter	Total population n = 2031	Rural area n = 1239 (61.0)	Urban area n = 792 (39.0)	p-value*
Age (years)	16.5 ± 1.2	16.4 ± 1.2	16.5 ± 1.2	0.121^a^
Sex, female (%)	1141 (56.2)	771 (62.2)	370 (46.7)	< 0.01^b^
FAS-score, high (%)	1317 (66.1)	815 (66.9)	502 (63.4)	0.377^b^
Non-smokers (%)	1396 (70.8)	856 (70.6)	540 (71.1)	0.999^b^
Healthy diet Score	2.0 ± 1.1	2.0 ± 1.1	2.0 ± 1.1	0.429^a^
Physical activity (min)	45.0 (30–60)	40.0 (25–60)	50.0 (30–80)	< 0.01^c^
BMI (kg/m^2^)	21.9 ± 3.5	21.8 ± 3.4	22.1 ± 3.7	0.024^a^
Systolic BP (mmHg)	122.7 (114.7–130.0)	121.3 (114.0–129.3)	123.7 (116.0–131.3)	< 0.01^c^
Diastolic BP (mmHg)	71.1 ± 7.6	71.0 ± 7.6	71.4 ± 7.7	0.096^a^
Total cholesterol (mg/dl)	157.0 (138.0–176.0)	158.0 (139.5–178.0)	156.0 (136.0–174.0)	< 0.01^c^
Fasting blood glucose (mg/dl)	76.6 ± 9.6	76.2 ± 9.4	77.3 ± 9.9	0.022^a^

Values are displayed as n (%), mean ± SD or median (IQR)

*FAS-Score* Family Affluence Score, *BMI* Body Mass Index, *BP* blood pressure

*p values are derived from ^a^Student´s t-test, ^b^Pearson-χ^2^-test and ^c^Mann–Whitney-U-Test

We observed no difference in SES between urban and rural teenagers as measured by the FAS (Table 2). Female sex was more prevalent in the rural area than in the urban area (62.2% vs. 46.7%, p < 0.01). When comparing the urban with the rural group average PA was higher in urban adolescents (50.0 min/day vs. 40.0 min/day, p < 0.01). Significant differences have also been observed for BMI, systolic BP, total cholesterol and fasting blood glucose. However, after adjustment for age and sex (generalized linear model) only the difference in mean daily PA between urban and rural adolescents remained significant (p < 0.01). The differences observed in univariate analysis were driven by unequal distribution of sex (systolic BP, total cholesterol and fasting blood glucose) or age (BMI, systolic BP, total cholesterol and fasting blood glucose) (Table 3).

**Table 3:** Impact of living in rural or urban areas on CVH metrics

Parameter	GLM		
	Parameters	B (SE)	p value
Physical activity	Rural (vs urban)	− 7.60 (1.87)	< 0.01
	Male sex (vs female)	18.57 (1.84)	< 0.01
	Age (years)	− 1.18 (0.78)	0.127
BMI	Rural (vs urban)	− 0.30 (1.60)	0.058
	Male sex (vs female)	1.14 (1.58)	0.472
	Age (years)	0.48 (0.07)	< 0.01
Systolic BP	Rural (vs urban)	− 0.43 (0.49)	0.376
	Male sex (vs female)	9.71 (0.48)	< 0.01
	Age (years)	0.63 (0.20)	< 0.01
Total cholesterol	Rural (vs urban)	1.39 (1.32)	0.292
	Male sex (vs female)	− 19.17 (1.29)	< 0.01
	Age (years)	1.72 (0.55)	< 0.01
Fasting blood glucose	In rural (vs urban)	− 0.32 (0.43)	0.460
	Male sex (vs female)	4.55 (0.43)	< 0.01
	Age (years)	− 0.45 (0.18)	0.012

*GLM* Generalized Linear Model, *BMI* Body Mass Index, *BP* blood pressure, *B* standardized regression coefficient

A detailed analysis of sex differences in the distribution of CVH metrics has been previously published [25].

## Discussion

In the present study we assessed CVH metrics in a healthy teenage population in the geographical region of North and South Tyrol in Austria and the northern part of Italy and analyzed differences between urban and rural areas. In a first step, we focused on ideal CVH which showed a generally low prevalence in the Tyrolean teenage population with no significant difference between urban and rural regions. Only 1.3% of urban teenagers met ideal criteria for all seven health metrics defined by the AHA compared to 1.7% of rural adolescents. Smoking and healthy diet were the health metrics most frequently categorized as poor with 29.4% for smoking in both urban and rural areas and 35.3% and 33.1% for healthy diet according to urban and rural areas. In addition, PA was categorized as non-ideal in 51.9% of urban and 61.6% of rural participants.

In a second step, mean values of CVH metrics were compared between urban and rural residents. After adjustment for age and sex in a generalized linear model only the difference in PA remained significant between these two groups with urban adolescents reporting of a ten minute longer period of moderate to vigorous-intensive activity per day than their rural counterparts.

Our results on ideal CVH in adolescents are comparable with data from the Healthy Lifestyle in Europe by Nutrition in Adolescence (HELENA-) cross-sectional study which was conducted in 9 European countries during 2007 and 2009. In this study there was also a low prevalence of ideal CVH metrics, especially regarding non-smoking (60.9%) and healthy diet (1.7%) [26]. Comparing ideal CVH in Tyrolean adolescents to participants from the National Health and Nutrition Examination Surveys in the U.S. they also show comparable results. Likewise, the number of study participants meeting criteria for all ideal 7 health metrics is very low with less than 2%. However, the participants in the mentioned U.S. study were aged between 20 and 65 years indicating that non-ideal CVH might continue into adulthood [27].

Regarding urban–rural differences in ideal CVH we could not observe any significant difference in the summary score of ideal CVH metrics. However, literature on ideal CVH reveals that urban areas have a more favorable risk factor profile than their rural counterparts. Two studies in adolescent cohorts reported that those living in urban communities during childhood and adolescence had a better cardiovascular risk factor profile and less subclinical markers of CVD, like intima-media thickness, left ventricular mass, arterial stiffness or endothelial dysfunction [11, 12]. Similarly, Nuotio et al. in the Cardiovascular Risk in Young Finns Study also showed that 9- to 18- year-old participants living in the urban area were physically more active and had significantly lower systolic BP, total cholesterol, LDL-cholesterol and triglyceride levels but were more likely to smoke at the age of 9- to 18 years than their rural peers. The more favorable cardiovascular risk factor profile of urban adolescents let to lower carotid artery intima media thickness, lower left ventricular mass and higher pulse wave velocity in adulthood in comparison to rural participants [14].

In 2018, Lawrence et al. found that young adults living in metropolitan areas exhibit more favorable CVH than individuals living in rural areas, and that population density largely accounts for this association. Better opportunities for PA as well as the social environment in dense areas might promote CVH in young adults [15].

It is a well-known fact that PA improves health. In 2018, Piercy et al. released an adapted version of the 2008 *U.S. Department of Health and Human Services Physical Activity Guidelines for Americans* recommending at least one hour of PA each day, containing aerobic, muscle- and bone strengthening exercise [28]. On average, the proposed PA levels were not reached in our cohort, neither in the urban nor in the rural comparative group. These outcomes are comparable to the results of other studies investigating the amount of PA among children and adolescents [29, 30]. Previous research suggested that rural residents may have less access to optimized built environments, making it harder to integrate PA into their daily life. The concept of the built environment covers a variety of contexts positively influencing the people’s ability to adopt a more active lifestyle, for instance including land use patterns to create parks and green spaces or the public transport system in order to reach sport facilities more easily [31]. Furthermore, social factors like family obligations and a lack of physically active peers may complement differences in the activity behavior of rural teenagers [32].

### Strengths and limitations

The large and homogenous study cohort consists of adolescents from all school types as well as apprentices from all regions of the study area. Data was collected recently in a time-period of two years by a small and stable study team consisting of medical specialists, medical students and research assistants. However, some limitations of the study should be considered. Classification in rural and urban might differ to other countries making it difficult to compare our results with those of other studies. Except for weekends and school holidays the indicated main residence might not correspond to the place where some pupils or apprentices spend most of the time during the week since they reside in a boarding or vocational school away from their home place. Thus, adolescents living in the rural area might also be exposed to urban influences during school time. In addition, PA was self-reported in a standardized interview conducted by trained staff. It has been shown that objectively measured PA in comparison to the self-reported one resulted in a lower level of ideal activity [26]. The latter limitation would affect adolescents living in urban as well as rural areas and we mainly focused on differences between the two groups and not on absolute values.

## Implications and contribution

In the current study, prevalence of ideal CVH for adolescents living in urban as well as rural areas was low. This result highlights the need for early health intervention, especially the categories smoking and healthy diet would benefit from improvement in both urban and rural areas. Ideal CVH was not significantly different in urban and rural areas, however we observed that urban adolescents showed significantly more PA. The difference in PA between urban and rural adolescents indicates that it is necessary to investigate regional differences before optimizing healthcare resources and improving prevention. Offering a wide range of team sport facilities independent of season and attractive to adolescents in rural areas may considerably contribute to an improvement of ideal PA. Long-term observation will be necessary in order to monitor developing differences of CVH in urban and rural areas at an early stage.

## Supplementary Information

**Additional file 1.** Additional Figure 1 displays the percentages of adolescents by number of ideal cardiovascular health metrics according to the place of living.

## Data Availability

The data that support the findings of this study are available from the corresponding author upon reasonable request.

## Abbreviations

AHA: American Heart Association
BMI: Body mass index
BP: Blood pressure
CVD: Cardiovascular disease
CVH: Cardiovascular health
DASH: Dietary approaches to stop hypertension
EVA: Early vascular aging
FAS: Family affluence score
SES: Socio-economic status
PA: Physical activity
