# Supplementary material for: Prevalence and differences of ideal cardiovascular health in urban and rural adolescents in the Region of Tyrol: results from the EVA Tyrol study
Source: BMC Cardiovasc Disord. 2021 Jul 13;21:338. doi: 10.1186/s12872-021-02156-6 (PMC8276470; doi:10.1186/s12872-021-02156-6)
Supplement: Supplementary file 1 — Additional file 1. Additional Figure 1 displays the percentages of adolescents by number of ideal cardiovascular health metrics according to the place of living. [file 12872_2021_2156_MOESM1_ESM.docx]

**Figure-Title: Fig 1. Prevalence of ideal cardiovascular health determinants in urban and rural adolescents.**


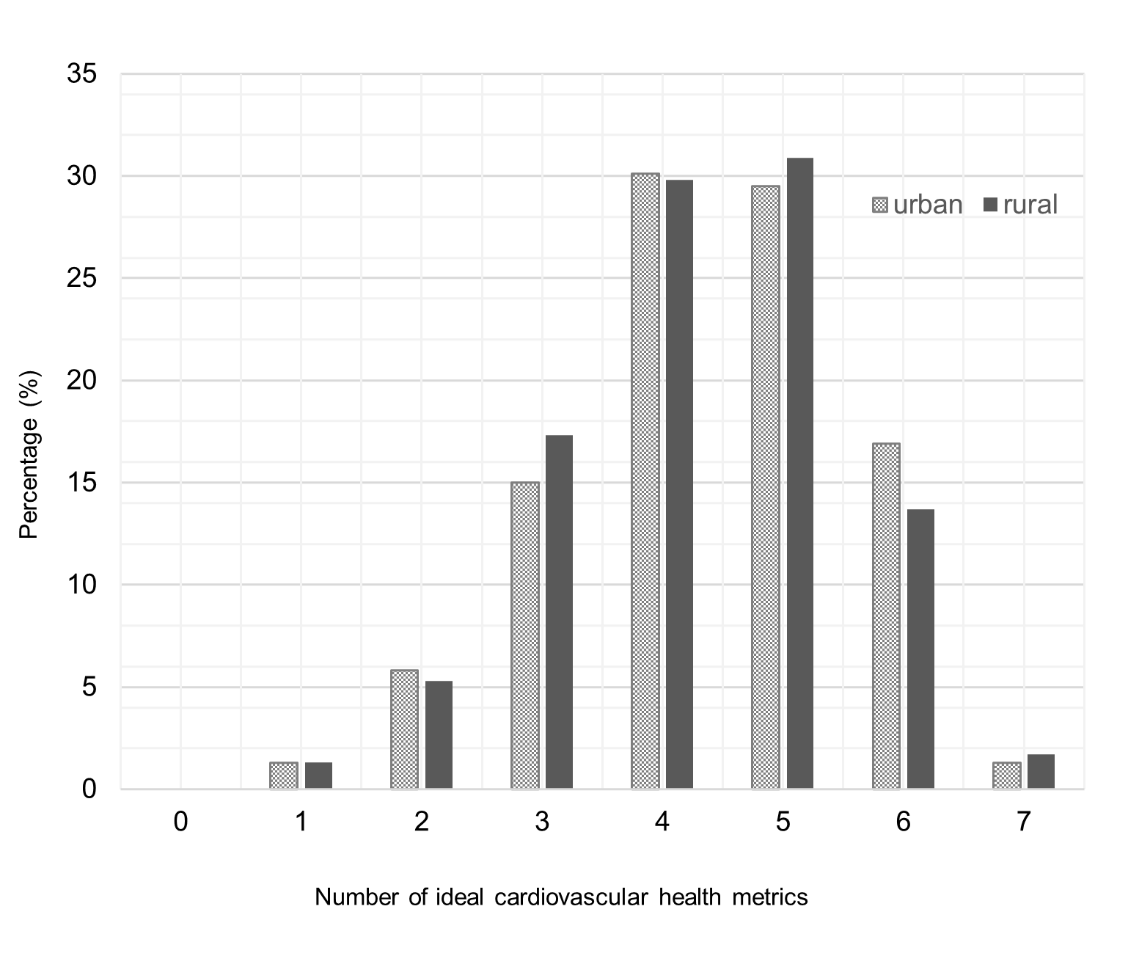


Figure-Caption: Figure 1 displays the percentages of adolescents by number of ideal cardiovascular health metrics according to the place of living.
